# Kaempferol Attenuates Myocardial Ischemic Injury* via* Inhibition of MAPK Signaling Pathway in Experimental Model of Myocardial Ischemia-Reperfusion Injury

**DOI:** 10.1155/2016/7580731

**Published:** 2016-03-21

**Authors:** Kapil Suchal, Salma Malik, Nanda Gamad, Rajiv Kumar Malhotra, Sameer N. Goyal, Uma Chaudhary, Jagriti Bhatia, Shreesh Ojha, Dharamvir Singh Arya

**Affiliations:** ^1^Department of Pharmacology, Cardiovascular Research Laboratory, All India Institute of Medical Sciences, New Delhi 110029, India; ^2^Department of Pharmacology, R.C. Patel Institute of Pharmaceutical Education and Research, Shirpur, Maharashtra 425405, India; ^3^Department of Biomedical Science, Bhaskaracharya College of Applied Science, University of Delhi, Delhi 110075, India; ^4^Department of Pharmacology and Therapeutics, College of Medicine and Health Sciences, United Arab Emirates University, P.O. Box 17666, Al Ain, Abu Dhabi, UAE

## Abstract

Kaempferol (KMP), a dietary flavonoid, has antioxidant, anti-inflammatory, and antiapoptotic effects. Hence, we investigated the effect of KMP in ischemia-reperfusion (IR) model of myocardial injury in rats. We studied male albino Wistar rats that were divided into sham, IR-control, KMP-20 + IR, and KMP 20* per se *groups. KMP (20 mg/kg; i.p.) was administered daily to rats for the period of 15 days, and, on the 15th day, ischemia was produced by one-stage ligation of left anterior descending coronary artery for 45 min followed by reperfusion for 60 min. After completion of surgery, rats were sacrificed; heart was removed and processed for biochemical, morphological, and molecular studies. KMP pretreatment significantly ameliorated IR injury by maintaining cardiac function, normalizing oxidative stress, and preserving morphological alterations. Furthermore, there was a decrease in the level of inflammatory markers (TNF-*α*, IL-6, and NF*κ*B), inhibition of active JNK and p38 proteins, and activation of ERK1/ERK2, a prosurvival kinase. Additionally, it also attenuated apoptosis by reducing the expression of proapoptotic proteins (Bax and Caspase-3), TUNEL positive cells, and increased level of antiapoptotic proteins (Bcl-2). In conclusion, KMP protected against IR injury by attenuating inflammation and apoptosis through the modulation of MAPK pathway.

## 1. Introduction

Myocardial infarction (MI) results from sudden obstruction of blood supply to a part of the heart resulting in ischemia and death of the affected cardiac tissue. Although rapid reperfusion of the affected myocardium is an important aim of MI therapy, reperfusion itself may result in cell death and tissue damage [1]. The mechanism of myocardial ischemia-reperfusion injury is very complex and includes generation of reactive oxygen species (ROS), calcium overload, activation of proinflammatory cytokines, apoptosis, neutrophil infiltration, and endothelial dysfunction [2]. Thus, inhibition of oxidative stress and apoptosis could be a potential target for the development of novel strategy for ischemic disease. Furthermore, reactive oxygen species generated due to ischemia-reperfusion (IR) injury also activates intracellular signaling pathways such as mitogen activated protein kinases (MAPKs) [3, 4]. The MAPK subfamilies stress kinases such as p38 and c-jun N-terminal kinase (JNK) which cause inflammation and apoptotic cell death and prosurvival kinase, that is, extracellular regulated kinase (ERK 1/2) which regulates myocyte differentiation and proliferation, promotes cell survival, and confers tissue protection [5, 6]. Studies have demonstrated that activation of ERK1/ERK2 and inhibition of p38/JNK protect the myocardium from IR injury by reducing oxidative stress and inflammation and by maintaining cytoskeletal architecture [1, 7]. Therefore, relative activity of these proapoptotic and prosurvival kinase pathways will decide whether cell shall survive or perish.

Kaempferol (KMP; 3,4′,5,7-tetrahydroxyflavone) is a naturally occurring polyphenolic compound, present in great amounts in tea, broccoli, propolis, and grapefruit [8]. It is very well known to possess antioxidant [9], anti-inflammatory [10], antiapoptotic [8], anticancer [11], neuroprotective [12], and antidiabetic properties [13]. Recently, an* in vitro* study, the protective role of KMP in anoxia/reoxygenation injury* via* the inhibition of oxidative stress and apoptosis, was demonstrated [14]. Furthermore, considerable evidence also shows that KMP exerts its anti-inflammatory and cardioprotective activities by modulating MAPK pathway [8, 15]. Thus, with the background that p38, JNK, and ERK1/ERK2 play important roles in IR injury and KMP has modulating effect on these proteins, we undertook this study to investigate whether KMP has cardioprotective effect in IR injury and if so, whether it is mediated through MAPK pathway.

## 2. Materials and Methods

### 2.1. Animals and Reagents

Adult male albino Wistar rats (150–200 g) were procured from Central Animal House Facility of All India Institute of Medical Sciences, New Delhi. All experiments were conducted according to guidelines outlined in Indian National Science Academy guidelines for use and care of experimental animals in research and approved by Institutional Animal Ethics Committee (717/IAEC/13). Rats were kept in polypropylene cages (4 rats/cage) under standard temperature (25 ± 2°C), relative humidity (60 ± 5%) with 12 h light/dark cycle. All rats had access to food (Ashirwad Industries Ltd., Chandigarh, India) and water* ad libitum*. KMP was obtained from Sigma Aldrich, USA, respectively. For administration, KMP was dissolved in 0.5% dimethyl sulfoxide (DMSO). All other chemicals used were of analytical grade.

### 2.2. Experimental Protocol

#### 2.2.1. Myocardial Ischemia-Reperfusion Model

A total of 36 rats were distributed into 4 groups.

*Group 1* (Sham; *n* = 8). 0.5% DMSO (2 mL/kg/day; i.p.) was administered to rats for a period of 15 days. On the 15th day, thread was passed beneath the left anterior descending (LAD) coronary artery but was not occluded.

*Group 2* (IR-Control; *n* = 10). 0.5% DMSO (2 mL/kg/day; i.p.) was administered to rats for a period of 15 days. On the 15th day, rats were subjected to LAD coronary artery ligation for 45 min and then reperfused for 60 min.

*Group 3* (KMP-20 + IR; *n* = 10). KMP (20 mg/kg/day; i.p) was administered to rats for a period of 15 days. On the 15th day, rats were subjected to LAD coronary artery ligation for 45 min and then reperfused for 60 min.

*Group 4* (KMP* per se*; *n* = 8). KMP (20 mg/kg/day; i.p.) was administered to rats for a period of 15 days. On the 15th day, thread was passed beneath the LAD coronary artery but was not occluded.

### 2.3. Measurement of Hemodynamic Parameters

Rats were anesthetized with pentobarbitone sodium (60 mg/kg; i.p.). The neck was opened with a ventral midline incision and tracheostomy was performed. The rats were ventilated with room air from a positive pressure respirator (TSE animal respirator, Germany). The left jugular vein was cannulated with polyethylene tube and 0.9% normal saline was infused through it. The right carotid artery was cannulated with heparinised cannula and then connected to pressure transducer (Gould Statham P231D, USA) for assessment of blood pressure [systolic arterial pressure (SAP), mean arterial pressure (MAP), and diastolic arterial pressure (DAP)] and heart rate (HR). Furthermore, left thoracotomy was performed to record left ventricular pressures [rate of contraction (+LV*dP*/*dt*); rate of relaxation (−LV*dP*/*dt*); and preload, i.e., left ventricular end diastolic pressure (LVEDP)] using Biopac system software BSL 4.0 MP36. After the completion of surgical procedure, blood was drawn from heart and rats were sacrificed with an overdose of pentobarbitone sodium (150 mg/kg; i.p.). Hearts were excised; rinsed in ice cold saline; and stored for biochemical, histopathological, and ultrastructural evaluation, terminal deoxynucleotide transferase dUTP nick end labeling (TUNEL) assay, immunohistochemistry (IHC), and western blot analysis. Serum was obtained by centrifuging blood at 5000 rpm and was further used to assess lactate dehydrogenase (LDH) and Creatine Kinase-MB (CK-MB) isoenzyme activities and to estimate interleukin-6 (IL-6) and tumor necrosis factor-*α* (TNF-*α*) levels.

### 2.4. Measurement of Biochemical Parameters

For biochemical estimation, heart tissues were removed from liquid nitrogen, brought to room temperature, and weighed. Tissue homogenate was prepared with 10 equal volumes of 0.1 M phosphate buffer (pH 7.4) and part of it was used for the estimation of malondialdehyde (MDA) level, a marker of lipid peroxidation and reduced glutathione (GSH). The remaining part of the homogenate was centrifuged at 5000 rpm to obtain supernatant for the measurement of superoxide dismutase (SOD) and catalase (CAT) enzyme activities.

#### 2.4.1. Estimation of MDA Level

MDA level in the tissue was measured by a method described by Ohkawa et al. [16]. In this method, tissue MDA was measured by its reactivity with thiobarbituric acid in acidic conditions to generate pink color adducts whose absorbance was read at 532 nm.

#### 2.4.2. Estimation of GSH Content

GSH content was quantified by a method described by Moron et al. [17]. This method is based on the development of yellow color when 5,5′-dithiobis(2-nitrobenzoic acid) (DTNB) is added to compounds containing sulfhydryl group like glutathione. The homogenate was centrifuged with equal parts of 10% tricarboxylic acid (TCA) at 5000 rpm for 10 min. The supernatant thus collected contains glutathione which has thiol (–SH) group that reacts with DTNB at pH 8.0 to produce a yellow colored ion whose concentration was measured at 412 nm.

#### 2.4.3. Estimation of SOD Enzyme Activity

The enzyme activity of SOD was assayed by evaluating the extent of inhibition of pyrogallol autoxidation at pH 8.4 [18].

#### 2.4.4. Estimation of CAT

CAT enzyme activity was estimated by measuring difference in H_2_O_2_ extinction per unit time, as described previously [19].

### 2.5. CK-MB and LDH Enzymes Activities

CK-MB isoenzyme and LDH enzyme activities were measured spectrophotometrically in serum using kits from Spinreact, Spain, and Logotech Private Limited, India, respectively.

### 2.6. Estimation of Serum TNF-*α* and IL-6 Levels

For the estimation of serum TNF-*α* and IL-6 levels, rat tumor necrosis factor alpha (TNF-*α*) (Diaclone Tepnel Company, UK) and Interleukin-6 (IL-6) (RayBiotech, Inc., Norcross, GA) kits were used and estimated according to the manufacturer's instructions.

### 2.7. Histopathological Examination

Heart tissues were fixed in 10% neutral buffered formalin and then embedded in paraffin. Later, tissue sections (5 *μ*m thick) were cut using microtome (Leica RM 2125, Germany). Each section was stained with hematoxylin and eosin (H&E) and evaluated by light microscope (Dewinter Technologies, Italy). At least, three hearts from each group were examined for histological examination and graded for the severity of changes using score on a scale of severe (+++); moderate (++); mild (+); and nil (−).

### 2.8. Ultrastructural Evaluation by Transmission Electron Microscopy (TEM)

Karnovsky's fixed tissue sections were washed in ice-chilled phosphate buffer (0.1 M, pH 7.4) and postfixed for 2 h in 1% osmium tetroxide in the phosphate buffer at 4°C. Then, sections were embedded in araldite CY212 to make tissue blocks. Ultrathin tissue sections (70−80 nm) were cut by ultramicrotome (Ultracut E, Reichert, Austria), stained with uranyl acetate and lead acetate to visualize them under transmission electron microscope (Morgagni268D, FeiCo., Netherlands).

### 2.9. Immunohistochemistry Studies

After sacrificing rats, heart tissues were immediately fixed in 10% neutral buffered formalin. 5 *μ*m thick sections were cut using microtome. Sections were deparaffinized in xylene and rehydrated in graded series of ethanol. Following this, antigen retrieval was performed by heating sections in a microwave at 95°C for 10 min in citrate buffer (10 mM; pH 6.0). To quench any endogenous peroxidase activity, sections were incubated with 30% hydrogen peroxide (H_2_O_2_) in methanol for 10 min. Furthermore, sections were blocked with normal goat serum for 1 h at room temperature to block any nonspecific binding. Then, sections were allowed to react with primary rabbit monoclonal antibody (mAb) against Bax (Santa Cruz, USA), Bcl-2 (Abcam, UK), and Caspase-3 (Cell signaling, USA) for 48 h followed by incubation with horse radish peroxidase- (HRP-) conjugated secondary antibodies (Merck Genei, India) for 2 h. Colorimetric reaction was performed with the addition of 3,3′-diaminobenzidine (DAB). Sections were then visualized and photographed under light microscope (Dewinter Technologies, Italy).

### 2.10. TUNEL Assay

ApoBrdu DNA fragmentation assay kit was used to analyze apoptosis in heart sections. For deparaffinization and rehydration, sections were passed through xylene and graded series of ethanol and water. Furthermore, tissue sections were incubated with Proteinase K and 30% H_2_O_2_ to enhance tissue permeability and to quench any endogenous peroxidase activity in the tissue sections, respectively. Following this, sections were incubated with complete labeling reaction buffer and antibody solution, each for 1 h and 30 min. The antigen-antibody interaction was visualized using DAB. Sections were then counterstained with hematoxylin and mounted with DPX to visualize under light microscope. At least 5 fields in each slide were looked for any TUNEL positive cells in each group. Histopathological, ultrastructural, IHC, and TUNEL evaluation was performed by an investigator blinded to the groups studied.

### 2.11. Western Blot Analysis

Heart tissues were removed from liquid nitrogen, thawed, and weighed. Tissue homogenate was prepared with RIPA buffer (150 mM NaCl, 10% Triton X-100, 0.5% sodium deoxycholate, 0.1% sodium dodecyl sulphate, and 50 mM Tris base), supplemented with protease inhibitor cocktail (Sigma Aldrich, USA). Then homogenate was centrifuged at 12000 rpm for 15 min at 4°C. Protein concentration was measured by the method described by Bradford [20]. Each well was loaded with equal amount of proteins (40 *μ*g) in a sodium dodecyl sulphate polyacrylamide gel electrophoresis (SDS-PAGE) and then transferred to nitrocellulose membrane. Following this, membrane was probed with primary antibodies, that is, ERK1/ERK2 (Cell signaling, USA), phospho-ERK1/ERK2 (Thr202/Tyr204; Cell signaling, USA), SAPK/JNK (Cell signaling, USA), phospho SAPK/JNK (Thr183/Tyr185; Cell signaling, USA), p38 (Abcam; UK); phospho-p38 (Santa Cruz, USA), NF*κ*Bp65 (Cell signaling, USA), and *β*-actin (Cell signaling, USA) overnight at 4°C. These primary antibodies were detected by adding HRP-conjugated secondary antibodies (Merck Genei, India) after incubating for 2 h at room temperature. Later, the bound antibodies were visualized with an enhanced chemiluminescence (ECL) (Thermo Fischer Scientific Inc., USA) kit and quantified by densitometric analysis.

### 2.12. Statistical Analysis

Data were analyzed by one-way analysis of variance (ANOVA) followed by post hoc Tukey-Kramer multiple comparison test using GraphPad software. All data are expressed as mean ± SEM. Difference with *p* < 0.05 were considered as statistically significant.

## 3. Results

### 3.1. Mortality

Due to bleeding or improper ligation of LADCA, the total of 8.34% mortality was observed in groups.

### 3.2. Effect on Cardiac Function

**Figure 1:**
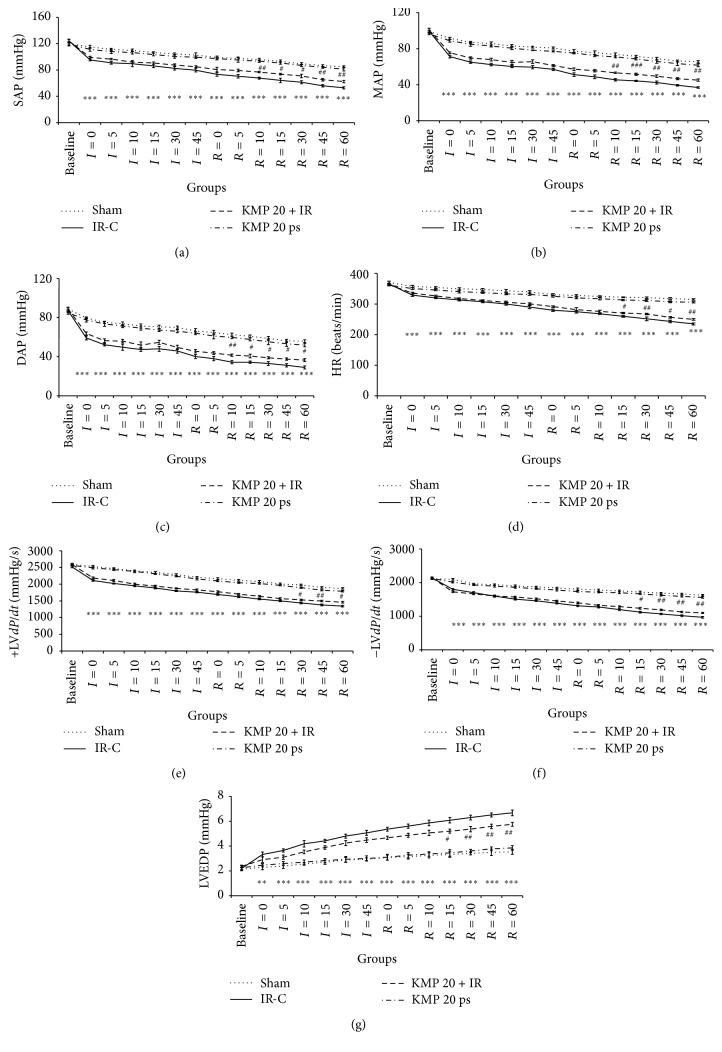
Effect of KMP on arterial pressure and ventricular function. (a) SAP; (b) MAP; (c) DAP; (d) HR; (e) maximal positive rate of the left ventricular pressure (+LV*dP*/*dt*_max_); (f) maximal negative rate of the left ventricular pressure (−LV*dP*/*dt*_max_); (g) LVEDP. IR-C: ischemia-reperfusion control; KMP 20 + IR: kaempferol 20 mg/kg/day + ischemia-reperfusion; KMP 20 ps: kaempferol 20 mg/kg/day* per se*. Data are expressed as the mean ± SEM; *n* = 6 in each group ^*∗∗∗*^ *p* < 0.001 versus sham; ^#^ *p* < 0.05, ^##^ *p* < 0.01, and ^###^ *p* < 0.001 versus IR-control.

To investigate the ability of KMP to improve cardiac functions, hemodynamic parameters (SAP, MAP, DAP, and HR) and ventricular functions (±LV*dP*/*dt* and LVEDP) were assessed in all the groups. Compared to sham group, IR injury resulted in hemodynamic impairment as demonstrated by significant (*p* < 0.001) reduction in arterial pressure (SAP, MAP, and DAP) and HR. Furthermore, IR injury exhibited ventricular dysfunction caused decline in ventricular contraction (+LV*dP*/*dt*) and relaxation (−LV*dP*/*dt*) with concomitant increase in preload (LVEDP) as compared to sham group (*p* < 0.001). Interestingly, KMP treatment (20 mg/kg) diminished detrimental effect of IR injury as it caused significant improvement in hemodynamic functions and preserved ventricular function (Figure 1).

### 3.3. KMP Reduced Lipid Peroxidation, Prevented Cardiac Injury Marker Release, and Restored Endogenous Antioxidants after IR

IR-challenged myocardium resulted in marked elevation of tissue MDA (*p* < 0.001), a marker of lipid peroxidation, and cardiac injury markers such as CK-MB and LDH in the serum (*p* < 0.001). IR injury also resulted in the depletion of antioxidants such as GSH, SOD (*p* < 0.001), and CAT (*p* < 0.01) in comparison to sham group. Intriguingly, KMP treatment markedly attenuated lipid peroxidation (*p* < 0.01), decreased consumption of antioxidants (*p* < 0.01), and prevented the release of cardiac injury markers into the serum (*p* < 0.01). This finding confirmed the antioxidant effect of KMP in myocardial IR injury (Table 1).

### 3.4. KMP Attenuated Apoptosis after IR

**Figure 2:**
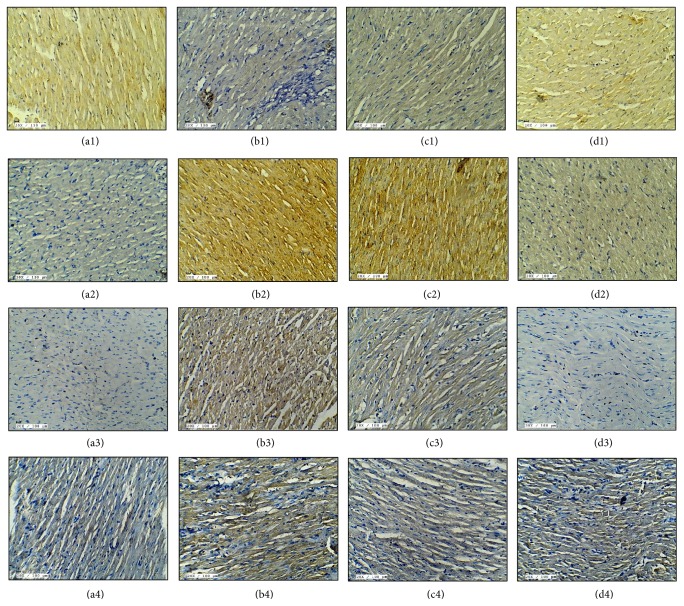
Effect of KMP on ((a1)–(d1)) Bcl-2 immunohistochemistry (20x; scale bar 100 *μ*m); ((a2)–(d2)) Bax immunohistochemistry (20x; scale bar 100 *μ*m); ((a3)–(d3)) Caspase-3 immunohistochemistry (20x; scale bar 100 *μ*m); ((a4)–(d4)) TUNEL positivity (20x; scale bar 100 *μ*m). ((a1)–(a4)) Sham; ((b1)–(b4)) ischemia-reperfusion control; ((c1)–(c4)) kaempferol 20 mg/kg/day + ischemia-reperfusion; ((d1)–(d4)) kaempferol 20 mg/kg/day* per se*.

We also verified the effect of KMP on myocardial apoptosis induced by IR injury. TUNEL assay was performed to detect DNA fragmentation in apoptotic nuclei. An increase in the number of TUNEL positive nuclei was observed in IR rat hearts. On the contrary, few TUNEL positive apoptotic nuclei were seen in KMP treatment group (Figure 2).

Further, to support the role of KMP on apoptosis, immunohistochemical analysis of regulatory proteins of apoptosis such as Bax, Caspase-3, and Bcl-2 was performed in all the groups. As anticipated, KMP treatment for 15 days decreased proapoptotic proteins (Bax and Caspase-3) and increased Bcl-2 expressions in IR-induced hearts (Figure 2).

### 3.5. KMP Decreased Inflammatory Markers after IR

**Figure 3:**
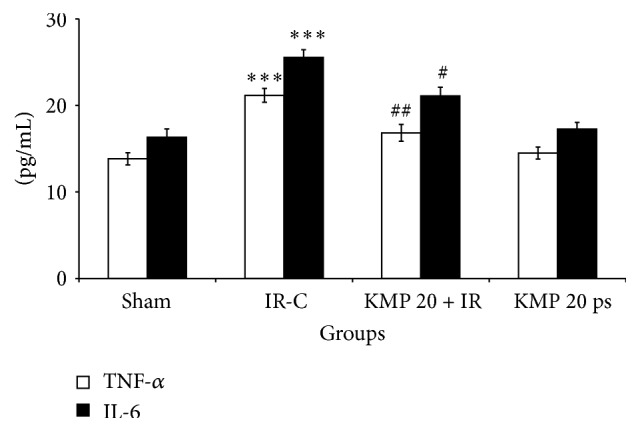
Effect of KMP on TNF-*α* and IL-6 levels. TNF-*α*: tumor necrosis factor alpha; IL-6: interleukin-6. IR-C: ischemia-reperfusion control; KMP 20 + IR: kaempferol 20 mg/kg/day + ischemia-reperfusion; KMP 20 ps: kaempferol 20 mg/kg/day* per se*. The values are expressed as mean ± SEM; *n* = 6 in each group ^*∗∗∗*^ *p* < 0.001 versus sham; ^#^ *p* < 0.05; ^##^ *p* < 0.01 versus IR-control.

Inflammatory reaction is an integral part of immune response to myocardial IR injury. Markers of inflammation such as TNF-*α* and IL-6 are usually present at undetected levels in normal heart but are upregulated in stress conditions such as myocardial IR injury. Likewise, we found significant increase in the levels of inflammatory cytokines (TNF-*α* and IL-6) in IR-control group (*p* < 0.001) as compared to sham group, while KMP treatment for 15 days significantly downregulated inflammatory markers (*p* < 0.05 for IL-6 and *p* < 0.01 for TNF-*α*) (Figure 3).

**Figure 4:**
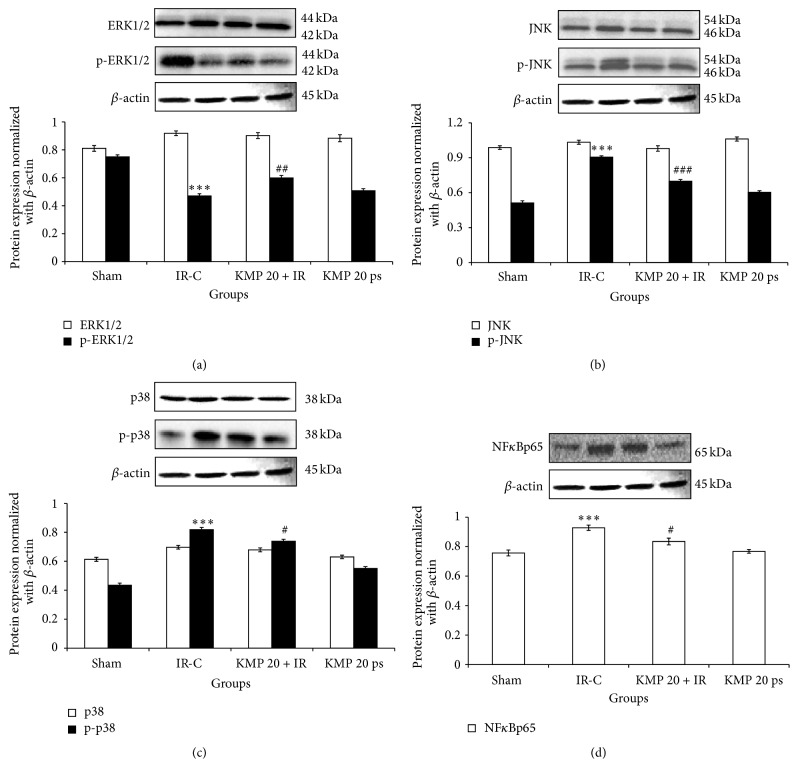
Effect of KMP on MAPKs protein expressions. (a) ERK1/ERK2, p-ERK1/ERK2; (b) JNK, p-JNK; (c) p38, p-p38; (d) NF*κ*Bp65. Data are expressed as normal intensity (% control). All the values are expressed as mean ± SEM; *n* = 3 per group. ^*∗∗∗*^ *p* < 0.001 versus sham; ^#^ *p* < 0.05; ^##^ *p* < 0.01; ^###^ *p* < 0.001 versus IR-control.

To further delineate the role of inflammation in IR injury, western blot analysis was performed to assess the expression of NF*κ*Bp65 in myocardium. In IR insulted myocardium, there was increased expression of NF*κ*Bp65 in myocardium, but KMP treatment significantly normalized the expression and ameliorated the inflammation (Figure 4).

### 3.6. KMP Normalized Protein Expressions after IR

Further, to delineate the mechanistic pathways of antiapoptotic and anti-inflammatory effects of KMP in IR injury, we measured ERK1/ERK2 and p38/JNK expressions which form an upstream signaling pathway in apoptotic and inflammatory reactions in the myocardium. In IR-challenged myocardium, there was downregulation of ERK1/ERK2 and activation of p38/JNK (*p* < 0.001) pathway, while KMP pretreatment significantly (*p* < 0.01) normalized their expressions and protected against IR injury (Figure 4).

### 3.7. KMP Recovered Myocardial Architecture after IR

**Figure 5:**
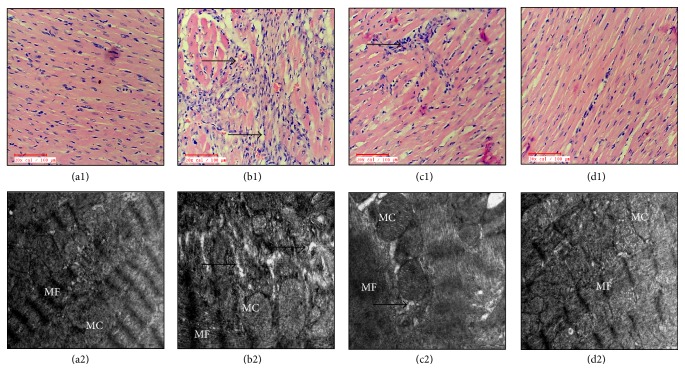
Effect of KMP on histopathological (20x; *n* = 3) and ultrastructural alterations (*n* = 3). ((a1)-(a2)) Sham; ((b1)-(b2)) ischemia-reperfusion control; ((c1)-(c2)) kaempferol 20 mg/kg/day + ischemia-reperfusion; ((d1)-(d2)) kaempferol 20 mg/kg/day* per se*. MF: myofibrils; MC: mitochondria.

Figure 5 illustrates the effect of KMP on morphological changes in IR insulted myocardium. Sham and* per se* groups exhibited normal myocardial architecture with no evidence of inflammation. IR injured myocardium displayed marked edema and membrane damage along with infiltration of inflammatory cells and higher histological score. Intriguingly, KMP treatment improved myonecrosis, preserved myocardial architecture, and exhibited a low histological injury score (Table 2).

On ultrastructural analysis, sham and* per se* groups demonstrated normal mitochondria and myofibrils, while IR-control rats revealed nuclear condensation, myonecrosis, mitochondrial swelling, and disruption of cristae with vacuolation in the myocardium. However, KMP treatment displayed lesser mitochondrial swelling and nuclear condensation without any necrotic changes (Figure 5).

## 4. Discussion

The present study reports novel finding related to the signaling pathways by which KMP exerts its cardioprotective activity in experimental model of IR. We have shown that KMP prevents the development and progression of IR injury and improves cardiac performance, primarily through the activation of ERK1/ERK2 and suppression of p38/JNK/TNF-*α*/NF-*κ*Bp65 pathway. Moreover, our study provides substantial evidence that KMP exerts potent antioxidant and antiapoptotic effects as inferred by reduced MDA levels and TUNEL positivity and upregulation of antiapoptotic markers. All these wide arrays of activities of KMP lead to improved contractile function and cardiac output.

It is well established that reperfusion of the ischemic tissue causes increased generation of oxygen rich-free radicals that disrupt balance between oxidants and antioxidants leading to uncontrolled myocardial injury. These free radicals cause lipid peroxidation, denature cellular proteins, and DNA. This results in the loss of membrane integrity and release of cardiac enzymes such as LDH and CK-MB from intracellular compartment to extracellular fluid [21, 22]. Hence, as predicted, there was reduction in levels of antioxidants such as GSH, SOD, and CAT which are endogenous free radical scavengers in IR-challenged myocardium. Interestingly, KMP treatment for 15 days bolstered the endogenous antioxidant defense system, preserved membrane integrity, and prevented release of CK-MB and LDH into the extracellular fluid. Various studies on KMP have established that KMP scavenges superoxide and reduces hydroxyl radical formation by chelating ferrous and cuprous ions in Fenton reaction besides increasing the expression or activities of SOD and CAT and preventing lipid peroxidation [9, 23]. Thus, it appears that KMP modulates the production of lipid peroxides and augments the overall antioxidant defense system in the myocardium, thereby protecting heart from ischemic injury. Moreover, evidence suggests that MAPK pathway is activated by ROS through the inactivation of MAPK phosphatases (MKPs). It should be noted that ERK is responsible for cell proliferation and cell survival, while p38 MAPKs/JNKs are involved in cell death and tight regulation of these pathways is paramount in determining cell fate [1, 4, 22]. In the present study, increased oxidative stress and p38 MAPKs/JNKs and decreased ERKs suggest increased cell death in IR-C rats, while the reverse effect in KMP treated group suggests cytoprotective effect of KMP through these pathways.

It is well known that Bax is activated by MAPK and is responsible for mediating cell apoptosis at the time of reperfusion. Also, the cardiac damage caused by apoptosis after IR is limited by the activation of ERK1/ERK2 [24]. Various studies have shown that the targeted inhibition of p38 MAPK reduced cardiomyocyte apoptosis and improved cardiac performance following IR injury [4, 22, 25]. Furthermore, melatonin protected against hepatic ischemia-reperfusion injury by inhibiting cell apoptosis which was mediated in part through inhibition of JNK and p38 MAPK signaling pathways [26]. In the present study, the greater activity of ERK1/ERK2, enhanced expression of Bcl-2 and attenuation of Bax, Caspase-3, and DNA fragmentation in KMP treated groups relative to untreated groups exhibits cell survival following IR insult. In line with this, Xiao et al. [8] showed that flavanol KMP protected against doxorubicin-induced cardiotoxicity by promoting cell survival. Thus, activation of ERK1/ERK2 and inhibition of p38/JNK pathways could be plausible mechanism contributing to its antioxidant and antiapoptotic effects.

A key role of inflammation has been reported in the pathogenesis of IR-induced myocardial injury. Recent studies have indicated that IR-induced oxidative stress and activation of p38 and JNK upregulate NF-*κ*B signaling followed by TNF-*α* production [27, 28]. In its inactive state, NF-*κ*B is sequestrated in the cytoplasm by an inhibitory protein I*κ*B. During IR injury, increased ROS activates IKK*β* (I*κ*B kinase) which in turn phosphorylates I*κ*B leading to the dissociation of I*κ*B from NF-*κ*B subunits. NF-*κ*B translocates to nucleus and induces expression of inflammatory cytokines such as TNF-*α* and IL-6 as well as various adhesion molecules [27, 29]. In line with this, we observed increased serum TNF-*α*/IL-6 levels and NF-*κ*Bp65 expressions in IR insulted myocardium. These molecular changes were further supported by inflammatory and necrotic changes in the histopathological examination of the tissue. There was marked edema, myonecrosis, and infiltration of inflammatory cells in IR-induced rats. Conversely, KMP treatment decreased the expressions of inflammatory markers and preserved normal morphological structure in the treated rats. A study by Luo et al. [10] provide* in vivo* evidence that KMP reduced inflammatory lesion in diabetes by reducing TNF-*α* and IL-6 levels along with the downregulation of IKK and subsequent inhibition of NF-*κ*B pathway activation. Thus, these findings suggest that KMP may prove to be a promising therapeutic agent for the attenuation of inflammation in myocardial IR injury.

Thus, our study demonstrated the efficiency of KMP in attenuating oxidative stress, apoptosis, and inflammation in IR injured myocardium* via* the upregulation of ERK1/ERK2 and downregulation of p38/JNK pathway. However, further studies are warranted to interpret its role in myocardial IR injury and to establish its clinical efficacy.

## Figures and Tables

**Table 1:** Effect of KMP on biochemical parameters.

Groups	MDA (nmole/g tissue)	GSH (*µ*mole/g tissue)	SOD (U/mg protein)	CAT (U/mg protein)	LDH (U/L)	CK-MB (U/L)
Sham	63.27 ± 2.50	1.15 ± 0.08	5.48 ± 0.39	6.63 ± 0.38	450.62 ± 28.85	414.12 ± 19.84
IR-C	99.97 ± 3.49^**∗∗∗**^	0.62 ± 0.04^**∗∗∗**^	2.94 ± 0.51^**∗∗**^	3.87 ± 0.42^**∗∗**^	742.04 ± 20.00^**∗∗∗**^	616.64 ± 21.16^**∗∗∗**^
KMP 20 + IR	79.04 ± 3.8^##^	0.94 ± 0.06^##^	5.3 ± 0.5^##^	5.91 ± 0.47^#^	589.58 ± 20.02^##^	513.18 ± 20.62^##^
KMP 20 ps	67.96 ± 2.81	1.06 ± 0.06	5.33 ± 0.33	6.17 ± 0.3	499.19 ± 20.86	466.41 ± 18.42

MDA: malondialdehyde; GSH: reduced glutathione; SOD: superoxide dismutase; CAT: catalase; LDH: lactate dehydrogenase; CK-MB: Creatine Kinase-MB isoenzyme. IR-C: ischemia-reperfusion control; KMP 20 + IR: kaempferol 20 mg/kg/day + ischemia-reperfusion; KMP 20 ps: kaempferol 20 mg/kg/day *per se*. The values are expressed as mean ± SEM; *n* = 6 in each group; ^*∗∗*^ *p* < 0.01, ^*∗∗∗*^ *p* < 0.001 versus sham; ^#^ *p* < 0.05; ^##^ *p* < 0.01 versus IR-control.

**Table 2:** Effect of KMP on histological scoring of cardiac tissue.

Groups	Necrosis	Edema	Inflammation
Sham	−	−	−
IR-C	++	++	+++
KMP 20 + IR	+	+	+
KMP 20 ps	−	−	−

(+++) severe; (++) moderate; (+) mild; (−) nil. IR-C: ischemia-reperfusion control; KMP 20 + IR: kaempferol 20 mg/kg/day + ischemia-reperfusion; KMP 20 ps: kaempferol 20 mg/kg/day *per se*.

## Abbreviations

ROS: Reactive oxygen species
IR: Ischemia-reperfusion
MAPKs: Mitogen activated protein kinases
JNK: c-Jun N-terminal kinase
ERK1/ERK2: Extracellular regulated kinase 1/kinase 2
KMP: Kaempferol
SAP: Systolic arterial pressure
MAP: Mean arterial pressure
DAP: Diastolic arterial pressure
±LV*dP*/*dt*: Rate of change of pressure development
LVEDP: Left ventricular end diastolic pressure
TUNEL: Terminal deoxynucleotide transferase dUTP nick end labeling
IHC: Immunohistochemistry
LDH: Lactate dehydrogenase
CK-MB: Creatine Kinase-MB isoenzyme
IL-6: Interleukin-6
TNF-*α*: Tumor necrosis factor-*α*
MDA: Malondialdehyde
GSH: Reduced glutathione
SOD: Superoxide dismutase
CAT: Catalase
TEM: Transmission electron microscopy
ANOVA: One-way analysis of variance.

## References

[1] Li C., He J., Gao Y., Xing Y., Hou J., Tian J. (2014). Preventive effect of total flavones of Choerospondias axillaries on ischemia/reperfusion-induced myocardial infarction-related MAPK signaling pathway. *Cardiovascular Toxicology*.

[2] Jennings R. B. (2013). Historical perspective on the pathology of myocardial ischemia/reperfusion injury. *Circulation Research*.

[3] Son Y., Cheong Y.-K., Kim N.-H., Chung H.-T., Kang D. G., Pae H.-O. (2011). Mitogen-activated protein kinases and reactive oxygen species: how can ROS activate MAPK pathways?. *Journal of Signal Transduction*.

[4] Gao X. F., Zhou Y., Wang D. Y., Lew K. S., Richards A. M., Wang P. (2015). Urocortin-2 suppression of p38-MAPK signaling as an additional mechanism for ischemic cardioprotection. *Molecular and Cellular Biochemistry*.

[5] Li D.-Y., Tao L., Liu H., Christopher T. A., Lopez B. L., Ma X. L. (2006). Role of ERK1/2 in the anti-apoptotic and cardioprotective effects of nitric oxide after myocardial ischemia and reperfusion. *Apoptosis*.

[6] Shimada K., Nakamura M., Ishida E., Kishi M., Konishi N. (2003). Roles of p38- and c-jun NH2-terminal kinase-mediated pathways in 2-methoxyestradiol-induced p53 induction and apoptosis. *Carcinogenesis*.

[7] Yu J., Wang L., Akinyi M. (2015). Danshensu protects isolated heart against ischemia reperfusion injury through activation of Akt/ERK1/2/Nrf2 signaling. *International Journal of Clinical and Experimental Medicine*.

[8] Xiao J., Sun G.-B., Sun B. (2012). Kaempferol protects against doxorubicin-induced cardiotoxicity in vivo and in vitro. *Toxicology*.

[9] Al-Numair K. S., Chandramohan G., Veeramani C., Alsaif M. A. (2015). Ameliorative effect of kaempferol, a flavonoid, on oxidative stress in streptozotocin-induced diabetic rats. *Redox Report*.

[10] Luo C., Yang H., Tang C. (2015). Kaempferol alleviates insulin resistance via hepatic IKK/NF-*κ*B signal in type 2 diabetic rats. *International Immunopharmacology*.

[11] Jo E., Park S. J., Choi Y. S., Jeon W., Kim B. (2015). Kaempferol suppresses transforming growth factor-*β*1–induced epithelial-to-mesenchymal transition and migration of A549 lung cancer cells by inhibiting Akt1-mediated phosphorylation of Smad3 at threonine-179. *Neoplasia*.

[12] Yang E.-J., Kim G.-S., Jun M., Song K.-S. (2014). Kaempferol attenuates the glutamate-induced oxidative stress in mouse-derived hippocampal neuronal HT22 cells. *Food and Function*.

[13] Al-Numair K. S., Veeramani C., Alsaif M. A., Chandramohan G. (2015). Influence of kaempferol, a flavonoid compound, on membrane-bound ATPases in streptozotocin-induced diabetic rats. *Pharmaceutical Biology*.

[14] Guo Z., Liao Z., Huang L., Liu D., Yin D., He M. (2015). Kaempferol protects cardiomyocytes against anoxia/reoxygenation injury via mitochondrial pathway mediated by SIRT1. *European Journal of Pharmacology*.

[15] Yoon H.-Y., Lee E.-G., Lee H. (2013). Kaempferol inhibits IL-1*β*-induced proliferation of rheumatoid arthritis synovial fibroblasts and the production of COX-2, PGE2 and MMPs. *International Journal of Molecular Medicine*.

[16] Ohkawa H., Ohishi N., Yagi K. (1979). Assay for lipid peroxides in animal tissues by thiobarbituric acid reaction. *Analytical Biochemistry*.

[17] Moron M. S., Depierre J. W., Mannervik B. (1979). Levels of glutathione, glutathione reductase and glutathione S-transferase activities in rat lung and liver. *Biochimica et Biophysica Acta (BBA)—General Subjects*.

[18] Marklund S., Marklund G. (1974). Involvement of the superoxide anion radical in the autoxidation of pyrogallol and a convenient assay for superoxide dismutase. *European Journal of Biochemistry*.

[19] Aebi H. (1984). Catalase in vitro. *Methods in Enzymology*.

[20] Bradford M. M. (1976). A rapid and sensitive method for the quantitation of microgram quantities of protein utilizing the principle of protein-dye binding. *Analytical Biochemistry*.

[21] Malik S., Sharma A. K., Bharti S. (2011). In vivo cardioprotection by pitavastatin from ischemic-reperfusion injury through suppression of IKK/NF-*κ*B and upregulation of pAkt-e-NOS. *Journal of Cardiovascular Pharmacology*.

[22] Yu D., Li M., Tian Y., Liu J., Shang J. (2015). Luteolin inhibits ROS-activated MAPK pathway in myocardial ischemia/reperfusion injury. *Life Sciences*.

[23] Calderón-Montaño J. M., Burgos-Morón E., Pérez-Guerrero C., López-Lázaro M. (2011). A review on the dietary flavonoid kaempferol. *Mini-Reviews in Medicinal Chemistry*.

[24] Wang H., Zhu Q., Ye P. (2012). Pioglitazone attenuates myocardial ischemia-reperfusion injury via up-regulation of ERK and COX-2. *BioScience Trends*.

[25] Thomas C. J., Ng D. C. H., Patsikatheodorou N. (2011). Cardioprotection from ischaemia-reperfusion injury by a novel flavonol that reduces activation of p38 MAPK. *European Journal of Pharmacology*.

[26] Zhou L., Zhao D., An H., Zhang H., Jiang C., Yang B. (2015). Melatonin prevents lung injury induced by hepatic ischemia-reperfusion through anti-inflammatory and anti-apoptosis effects. *International Immunopharmacology*.

[27] Ma L., Liu H., Xie Z. (2014). Ginsenoside Rb3 protects cardiomyocytes against ischemia-reperfusion injury via the inhibition of JNK-mediated NF-*κ*B pathway: a mouse cardiomyocyte model. *PLoS ONE*.

[28] Wang W., Tang L., Li Y., Wang Y. (2015). Biochanin A protects against focal cerebral ischemia/reperfusion in rats via inhibition of p38-mediated inflammatory responses. *Journal of the Neurological Sciences*.

[29] Ramachandran S., Liaw J. M., Jia J. (2012). Ischemia-reperfusion injury in rat steatotic liver is dependent on NF*κ*B P65 activation. *Transplant Immunology*.

