# Corrigendum to “Kaempferol Attenuates Myocardial Ischemic Injury via Inhibition of MAPK Signaling Pathway in Experimental Model of Myocardial Ischemia-Reperfusion Injury”

**DOI:** 10.1155/2020/4530278

**Published:** 2020-06-18

**Authors:** Kapil Suchal, Salma Malik, Nanda Gamad, Rajiv Kumar Malhotra, Sameer N. Goyal, Uma Chaudhary, Jagriti Bhatia, Shreesh Ojha, Dharamvir Singh Arya

**Affiliations:** ^1^Department of Pharmacology, Cardiovascular Research Laboratory, All India Institute of Medical Sciences, New Delhi 110029, India; ^2^Department of Pharmacology, R.C. Patel Institute of Pharmaceutical Education and Research, Shirpur, Maharashtra 425405, India; ^3^Department of Biomedical Science, Bhaskaracharya College of Applied Science, University of Delhi, Delhi 110075, India; ^4^Department of Pharmacology and Therapeutics, College of Medicine and Health Sciences, United Arab Emirates University, P.O. Box 17666, Al Ain, Abu Dhabi, UAE

In the article titled “Kaempferol Attenuates Myocardial Ischemic Injury via Inhibition of MAPK Signaling Pathway in Experimental Model of Myocardial Ischemia-Reperfusion Injury” [1], it was identified that the beta-actin panels in Figures 4(a), 4(b), and 4(c) were identical. With the agreement of the editor, a revised version of Figure 4 is being provided with the data from a repeat of the experiment. The authors apologize for this error in the original publication. The corrected version of figure 4 is shown as Figure 1.

## Figures and Tables

**Figure 1:**
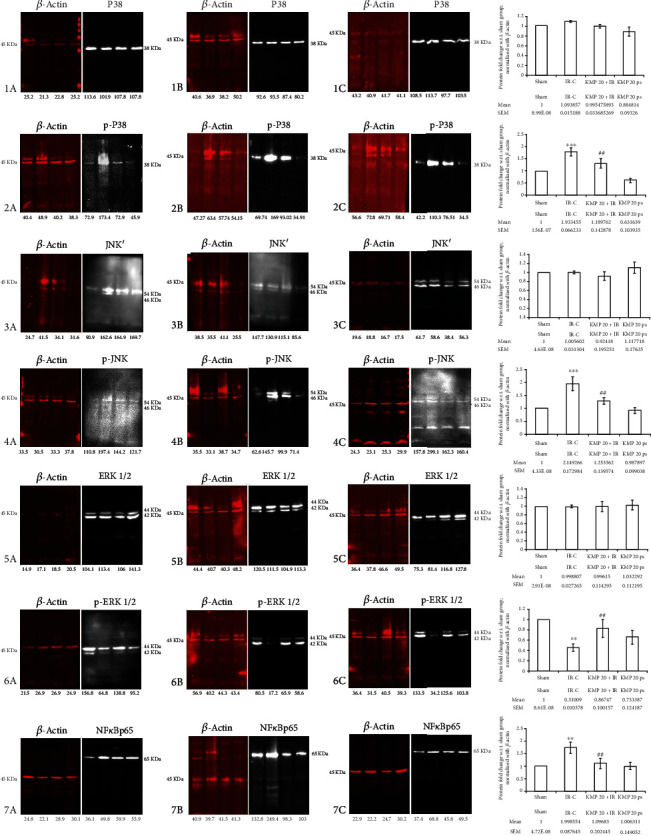
Effect of KMP on MAPKs protein expressions. (a) ERK1/ERK2, p-ERK1/ERK2; (b) JNK, p-JNK; (c) p38, p-p38; and (d) NF*κ*Bp65. Data are expressed as normal intensity (% control). All the values are expressed as mean ± SEM; *n* = 3 per group. ^∗∗∗^*p* < 0.001 versus sham; ^#^*p* < 0.05; ^##^*p* < 0.01; and ^###^*p* < 0.001 versus IR-control.
